# Characterization of Nonphysician Health Care Workers’ Burnout and Subsequent Changes in Work Effort

**DOI:** 10.1001/jamanetworkopen.2021.21435

**Published:** 2021-08-20

**Authors:** Liselotte N. Dyrbye, Brittny Major-Elechi, Prabin Thapa, J. Taylor Hays, Cathryn H. Fraser, Steven J. Buskirk, Colin P. West

**Affiliations:** 1 Mayo Clinic Program on Physician Well-Being, Mayo Clinic, Rochester, Minnesota; 2 Department of Health Sciences Research, Mayo Clinic, Rochester, Minnesota; 3 Mayo Clinic Leadership and Workforce Development, Mayo Clinic, Rochester, Minnesota; 4 Mayo Clinic, Rochester, Minnesota; 5 Department of Radiation Oncology, Mayo Clinic, Jacksonville, Florida

## Abstract

**Importance:**

Burnout is a pervasive, unrelenting problem among health care workers (HCWs), with detrimental impact to patients. Data on the impact of burnout on workforce staffing are limited and could help build a financial case for action to address system-level contributors to burnout.

**Objective:**

To explore the association of burnout and professional satisfaction with changes in work effort over 24 months in a large cohort of nonphysician HCWs.

**Design, Setting, and Participants:**

This longitudinal cohort study was conducted in Rochester, Minnesota; Scottsdale and Phoenix, Arizona; Jacksonville, Florida; and community-based hospitals and health care facilities in the Midwest among nonphysician HCWs who responded to 2 surveys from 2015 to 2017. Analysis was completed November 25, 2020.

**Exposures:**

Burnout, as measured by 2 items from the Maslach Burnout Inventory, and professional satisfaction.

**Main Outcomes and Measures:**

The main outcome was work effort, as measured in full-time equivalent (FTE) units, recorded in payroll records.

**Results:**

Data from 26 280 responders (7293 individuals aged 45-54 years [27.8%]; 20 263 [77.1%] women) were analyzed. A total of 8115 individuals (30.9%) had worked for the organization more than 15 years, and 6595 individuals (25.1%) were nurses. After controlling for sex, age, duration of employment, job category, baseline FTE, and baseline burnout, overall burnout (odds ratio [OR], 1.53; 95% CI, 1.38-1.70; *P* < .001), high emotional exhaustion at baseline (OR, 1.54; 95% CI, 1.39-1.71; *P* < .001), and high depersonalization at baseline (OR, 1.40; 95% CI, 1.21-1.62; *P* < .001) were associated with an HCW reducing their FTE over the following 24 months. Conversely, satisfaction with the organization at baseline was associated with lower likelihood of reduced FTE (OR, 0.73; 95% CI, 0.65-0.83; *P* < .001). Findings were similar when emotional exhaustion (OR per 1-point increase, 1.12; 95% CI, 1.10-1.16; *P* < .001), depersonalization (OR per 1-point increase, 1.10; 95% CI, 1.06-1.14; *P* < .001) and satisfaction with the organization (OR per 1-point increase, 0.83; 95% CI, 0.79-0.88; *P* < .001) were modeled as continuous measures. Nurses represented the largest group (1026 of 1997 nurses [51.4%]) reducing their FTE over the 24 months.

**Conclusions and Relevance:**

This cohort study found that burnout and professional satisfaction of HCWs were associated with subsequent changes in work effort over the following 24 months. These findings highlight the importance of addressing factors contributing to high stress among all HCWs as a workforce retention and cost reduction strategy.

## Key Points

**Question:**

Are burnout and professional satisfaction associated with changes in work effort among nonphysician health care workers (HCWs)?

**Findings:**

In this cohort study of 26 280 nonphysician HCWs using administrative and payroll records, levels of burnout and satisfaction were associated with changes in work effort over the ensuing 24 months.

**Meaning:**

These findings suggest that, given the critical importance of appropriate staffing and costs associated with hiring and training HCWs, efforts to mitigate burnout and increase professional satisfaction among HCWs should be part of workforce retention and cost reduction strategies.

## Teaser

This cohort study examines the associations of burnout and professional satisfaction with changes in work effort among nonphysician health care workers.

## Introduction

Burnout, an occupational phenomenon recognized by the World Health Organization as a result of chronic workplace stress,^1^ is common among health care workers (HCWs), and an indicator that “the nation’s health care system is failing to achieve the aims for system-wide improvement,” according to the National Academies of Sciences, Engineering, and Medicine.^2^ A substantial body of literature supports that burnout is associated with detrimental outcomes among HCWs and for the quality of care provided to patients.^2,3,4,5,6,7,8,9,10,11^ Factors that may contribute to burnout, such as excessive workload, administrative burdens, inadequate technology usability, constrained flexibility, and poor leadership, may also erode professional satisfaction.

Burnout and declines in professional satisfaction may also place additional strains on the health care system trying to meet the increasing demands for medical care in the setting of workforce shortages.^12,13,14,15,16^ Previous cross-sectional studies of physicians, nurses, and advance practice clinicians have demonstrated associations of burnout and satisfaction with intent to reduce work hours or leave their current job.^17,18,19,20,21,22,23,24,25,26^ A longitudinal study of physicians reported that burnout and satisfaction with the organization (employer) were associated with reductions in work effort, as measured by payroll records, over the following 2 years.^14^ Whether this association also holds true for other HCWs remains unexplored, to our knowledge. Research evaluating the implications of nonphysician HCW burnout and professional satisfaction has been identified as a priority area by the National Academies of Sciences, Engineering, and Medicine.^2^ To meet this need, we conducted a prospective cohort study of nonphysician HCWs to explore the association of burnout and professional satisfaction with changes in work effort over 24 months, as measured in full-time equivalent (FTE) units from payroll records.

## Methods

This was approved by the Mayo Clinic Institutional Review Board. Completion of the survey was voluntary and consent was implied upon completing the survey, and further consent was not required, pet the Mayo Clinic Institutional Review Board. This study is reported following the Strengthening the Reporting of Observational Studies in Epidemiology (STROBE) reporting guideline.

### Participants

As previously described,^27^ in October 2015 and 2017, all employees at Mayo Clinic (Rochester, Minnesota; Scottsdale and Phoenix, Arizona; Jacksonville, Florida; and community-based hospitals and health care facilities in the Midwest) received an email with a link to a survey administered by an external consulting company. For this analysis, the sample was limited to HCWs who were nonphysician health care professionals (eg, nurses, certified registered nurse anesthetists, physical therapists, occupational therapists, pharmacists, paramedics, social workers, nurse practitioners, and physician assistants), administrative office support workers, business professionals, clinical office support workers (ie, appointment specialists, desk attendants, registration coordinators, medical practice secretaries, coders, and unit coordinators), service and support workers (ie, patient care assistants, medical assistants, environmental service workers, housekeeping personnel, laundry personnel, general service personnel, security personnel, and telephone operators), and technicians and technologists (ie, surgical technologists, pharmacy technologists, dispatchers, laboratory technicians, research technicians, and radiology technologists) who remained employed at the organization and responded to 2 surveys, in 2015 and 2017. In 2015, 26 292 of 31 255 HCWs (84.1%) responded to the survey. Two years later, 26 303 of 31 158 (84.4%) HCWs responded to the 2017 survey. Of these responders, 26 280 HCWs completed both surveys and were included in this analysis.

Survey completion was voluntary, and all data were confidential. The external survey consulting company linked survey responses to organization-provided employee demographics (ie, sex, age), job category, and duration of employment. Only deidentified data were forwarded by the external consulting company to the statisticians (B.M.-E. and P.T.) for analysis.

### Work Effort

Administrative and payroll records were used to collect work effort, as measured in FTE units, for all nonphysician HCWs at the organization at both 2015 and 2017 time points. This information was provided by the organization to the external consulting company that linked the information to the survey responses.

### Burnout

The survey included 2 single-item measures from the Maslach Burnout Inventory (MBI) to measure emotional exhaustion and depersonalization, core components of professional burnout. As in other studies of HCWs,^28^ we considered individuals with a high score (ie, endorsed a frequency of once or more per week) on either item to have symptoms of burnout.^29,30^ Although the full 22-item MBI is the criterion standard for measuring burnout symptoms, we chose the 2 single items to reduce responder burden and because the 2 items have strong validity data. For example, the 2 single items stratify the risk of burnout.^29,30^ The single item of emotional exhaustion has an area under the receiver operating characteristic curve of 0.94 and a positive likelihood ratio of 14.9 compared with the emotional exhaustion subscale of the MBI.^29,30^ The single item of depersonalization has an area under the receiver operating characteristic curve of 0.93 and a positive likelihood ratio of 23.4 compared with the depersonalization subscale of the MBI.^29,30^

### Professional Satisfaction

The survey also included an item assessing professional satisfaction used previously in other studies.^31^ Respondents were asked to respond to the item, “Considering everything, how would you rate your overall satisfaction with Mayo Clinic as a whole at the present time?” using a 5-point Likert scale, with 1 indicating very dissatisfied and 5, very satisfied. We considered individuals who indicated they were very satisfied or satisfied to be satisfied with the organization.

### Statistical Analysis

Summary descriptive statistics were calculated. Bivariate analyses were conducted to explore differences between HCWs who did vs did not reduce their work effort. We conducted multivariable analysis to explore the association of change in FTE and baseline (2015) overall burnout, high emotional exhaustion, high depersonalization, and professional satisfaction after adjusting for age, sex, length of employment, job category, and baseline FTE. We repeated the analysis using emotional exhaustion, depersonalization, and professional satisfaction as continuous measures. Lastly, we repeated all multivariable analyses including the interaction term between age and sex. This did not produce statistically significant interaction results or meaningfully alter other estimates, and are not reported further. All analyses were performed using SAS statistical software version 9.4 (SAS Institute). A 2-sided *P* value of .05 was considered statistically significant. Analyses were completed on November 25, 2020.

## Results

Among 26 280 participants in the cohort, 20 263 (77.1%) were women, 7293 (27.8%) were aged 45 to 54 years, most had been employed less than 5 years (8570 individuals [32.6%]) or more than 15 years (8115 individuals [30.9%]), and one-quarter were nurses (6595 individuals [25.1%]) (Table 1). For both the 2015 and 2017 surveys, responders were different from nonresponders with respect to age, length of employment, job category, and assigned work effort (eTable 1 in the Supplement). The largest differences included more full-time employees and business professionals among responders (eTable 1 in the Supplement). There was no difference between responders and nonresponders to both surveys by sex.

**Table 1.** Demographic Characteristics at Baseline and Reduction in Work Effort Over 24 Months Among Nonphysician Health Care Workers

Characteristic	No. (%)			*P* value
	Overall	Reduced work effort	Did not reduce work effort	
Age, y				
<35	6672 (25.4)	843 (12.6)	5829 (87.4)	<.001
35-44	6540 (24.9)	399 (6.1)	6141 (93.9)	
45-54	7293 (27.8)	354 (4.9)	6939 (95.1)	
55-64	5451 (20.7)	368 (6.8)	5083 (93.2)	
≥65	323 (1.2)	33 (10.2)	290 (89.9)	
Missing	1	0	1	
Sex				
Women	20263 (77.1)	1792 (8.8)	18471 (91.2)	<.001
Men	6017 (22.9)	205 (3.4)	5812 (96.6)	
Missing	0	0	0	
Duration of employment, y				
<5	8570 (32.6)	843 (9.8)	7727 (90.2)	<.001
6-10	5325 (20.3)	422 (7.9)	4903 (92.1)	
11-15	4269 (16.2)	262 (6.1)	4007 (93.9)	
>15	8115 (30.9)	470 (5.8)	7645 (94.2)	
Missing	1	0	1	
Job category^a^				
Nurse	6595 (25.1)	1026 (15.6)	5569 (84.4)	<.001
Administrative office support	2966 (11.3)	99 (3.3)	2867 (96.7)	
Business professionals	4951 (18.8)	73 (1.5)	4878 (98.5)	
Clinical office support	2884 (11.0)	142 (4.9)	2742 (95.1)	
Health care professional	2423 (9.2)	165 (6.8)	2258 (93.2)	
Service and support personnel	2965 (11.3)	269 (9.1)	2696 (90.9)	
Technician or technologist	3496 (13.3)	223 (6.4)	3273 (93.6)	
Missing	3	0	3	

^a^ Clinical office support included appointment specialists, desk attendants, registration coordinators, medical practice secretaries, coders, and unit coordinators. Other health care professionals included certified registered nurse anesthetists, physical therapists, occupational therapists, pharmacists, paramedics, social workers, nurse practitioners, and physician assistants. Service and support personnel included patient care assistants, medical assistants, environmental service personnel, housekeeping personnel, laundry personnel, general service personnel, security personnel, and telephone operators. Technicians and technologists included surgical technologists, pharmacy technologists, dispatchers, laboratory technicians, research technicians, and radiology technologist.

At baseline 5695 of 26 023 respondents (21.9%) had high emotional exhaustion, 2389 of 25 996 respondents (9.2%) had high depersonalization, and 6177 of 25 906 respondents (23.8%) had overall burnout at baseline. Of 26 108 HCWs who rated their satisfaction at baseline, most HCWs were very satisfied (9125 respondents [35.0%]) or satisfied (13 339 respondents [51.1%]) with the organization, while fewer HCWs were neither satisfied nor dissatisfied (2556 respondents [9.8%]), dissatisfied (938 respondents [3.6%]), or very dissatisfied (150 respondents [0.6%]).

Among respondents, 1997 (8.2%) reduced their work effort between 2015 and 2017. Most respondents who reduced their work effort over the 24-month period were younger than 35 years (843 respondents [42.2%]), women (1792 respondents [89.7%]), and had less than 5 years of employment (843 respondents [42.4%]) (Table 1). More than half of respondents who reduced their work effort were nurses (1026 of 1997 respondents [51.4%]), representing 15.6% of all nurses in the cohort. Among those who did reduce their work effort, the mean (SD) reduction in work effort was 21.0% (17.9%), and among nurses specifically, mean (SD) work effort reduction was 18.7% (16.5%).

### Burnout, Satisfaction, and Reduction in Work Effort

Respondents who had burnout in 2015 were more likely to reduce their work effort over 24 months than those without burnout (644 of 6135 respondents [10.5%] vs 1326 of 19 771 respondents [6.7%]; *P* < .001). The attributable risk of 3.8% observed in this study suggests that of 1997 HCWs who reduced their work effort, approximately 75 did so because of burnout. Within this group, we estimate that approximately 38 nurses reduced their work effort because of burnout. The associations of emotional exhaustion, depersonalization, and satisfaction with the organization at baseline with reduction in work effort 24 months later are shown in the Figure. Increased frequencies of emotional exhaustion and depersonalization symptoms were associated with greater prevalence of reduction in work effort. Similarly, lower satisfaction with the organization was associated with higher prevalence of reduction in work effort.

**Figure.**
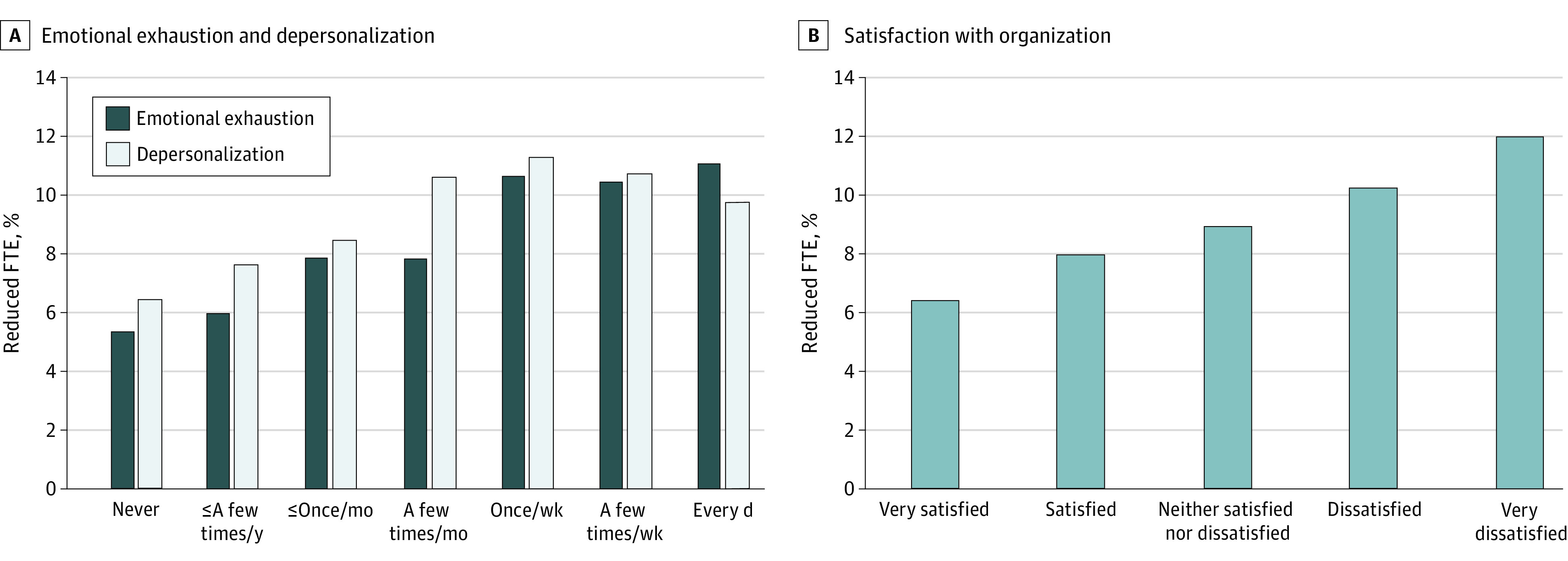
Emotional Exhaustion, Depersonalization, and Satisfaction With the Organization at Baseline and Reduction in Work Effort at 24 Months Work effort was measured by reduction in full time equivalent (FTE).

After controlling for sex, age, duration of employment, job category, site, and baseline FTE, overall burnout at baseline was associated with reduction in work effort over 24 months (OR, 1.53; 95% CI, 1.38-1.70; *P* < .001) (Table 2). Similarly, on multivariable analysis, high emotional exhaustion and high depersonalization at baseline were associated with reduction in work effort over 24 months (emotional exhaustion: OR, 1.54; 95% CI 1.39-1.71; *P* < .001; depersonalization: OR, 1.40; 95% CI, 1.21-1.62; *P* < .001).

**Table 2.** Multivariable Analysis Exploring Association of Health Care Workers’ Baseline Burnout, High Emotional Exhaustion, and High Depersonalization With Subsequent Reduction in Work Effort

Variable	Odds ratio (95% CI)	*P* value	Overall *P* value
**Burnout**			
Burnout at baseline^a^	1.53 (1.38-1.70)	NA	<.001
Men^b^	0.46 (0.40-0.54)	NA	<.001
Age, y			
<35	1 [Reference]	NA	<.001
35-44	0.57 (0.50-0.66)	<.001	
45-54	0.48 (0.41-0.56)	<.001	
55-64	0.67 (0.57-0.78)	<.001	
≥65	1.47 (0.99-2.19)	.06	
Length of employment, y			
≤5	1 [Reference]	NA	<.001
6-10	0.89 (0.78-1.01)	.07	
11-15	0.78 (0.67-0.92)	.002	
>15	0.76 (0.65-0.89)	<.001	
Job category			
Nurse	1 [Reference]	NA	<.001
Administrative office support	0.17 (0.14-0.21)	<.001	
Business professional	0.09 (0.07-0.12)	<.001	
Clinical office support	0.24 (0.20-0.29)	<.001	
Health care professional	0.39 (0.33-0.47)	<.001	
Service and support personnel	0.55 (0.47-0.64)	<.001	
Technician or technologist	0.34 (0.29-0.40)	<.001	
Baseline FTE, per 0.10-point increase^c^	1.17 (1.13-1.21)	NA	<.001
**Emotional exhaustion**			
High emotional exhaustion at baseline^a^	1.54 (1.39- 1.71)	NA	<.001
Men^b^	0.46 (0.40-0.54)	NA	<.001
Age, y			
<35	1 [Reference]	NA	<.001
35-44	0.58 (0.50-0.66)	<.001	
45-54	0.48 (0.41-0.56)	<.001	
55-64	0.67 (0.57-0.79)	<.001	
≥65	1.46 (0.98-2.17)	.06	
Length of employment, y			
≤5	1 [Reference]	NA	.001
6-10	0.89 (0.78-1.01)	.07	
11-15	0.78 (0.66-0.91)	.002	
>15	0.76 (0.65-0.88)	.001	
Job category			
Nurse	1 [Reference]	NA	<.001
Administrative office support	0.17 (0.14-0.21)	<.001	
Business professional	0.09 (0.07-0.12)	<.001	
Clinical office support	0.25 (0.20-0.30)	<.001	
Health care professional	0.39 (0.33-0.47)	<.001	
Service and support personnel	0.55 (0.47-0.63)	<.001	
Technician or technologist	0.35 (0.30-0.40)	<.001	
Baseline FTE, per 0.10-unit increase^c^	1.17 (1.13-1.21)	NA	<.001
**Depersonalization**			
High depersonalization at baseline^a^	1.40 (1.21-1.62)	NA	<.001
Men^b^	0.46 (0.39-0.53)	NA	<.001
Age, y			
<35	1 [Reference]	NA	<.001
35-44	0.57 (0.50-0.65)	<.001	
45-54	0.47 (0.40-0.55)	<.001	
55-64	0.65 (0.56-0.77)	<.001	
≥65	1.46 (0.99-2.16)	.06	
Length of employment, y			
≤5	1 [Reference]	NA	.002
6-10	0.9 (0.79-1.02)	.10	
11-15	0.78 (0.67-0.92)	.03	
>15	0.77 (0.66-0.90)	<.001	
Job category			
Nurse	1 [Reference]	NA	<.001
Administrative office support	0.17 (0.14-0.22)	<.001	
Business professional	0.09 (0.07-0.12)	<.001	
Clinical office support	0.24 (0.20-0.29)	<.001	
Health care professional	0.39 (0.33-0.47)	<.001	
Service and support personnel	0.55 (0.47- 0.64)	<.001	
Technician or technologist	0.35 (0.30-0.41)	<.001	
Baseline FTE, per 0.10-point increase	1.18 (1.14-1.22)	NA	<.001

Abbreviations: FTE, full-time equivalent; NA, not applicable.

^a^ Compared with no at baseline.

^b^ Compared with women.

^c^ Work effort was measured in FTE units recorded in payroll records.

A dose-response association was also found between reduced FTE and each 1-point worsening of exhaustion (OR, 1.12, 95% CI 1.10- 1.16, *P* < .001) and 1-point worsening of depersonalization (OR, 1.10; 95% CI, 1.06- 1.14; *P* < .001) between 2015 and 2017, after controlling for age, sex, length of employment, baseline FTE, job category, and site (eTable 2 in the Supplement).

In contrast, HCWs who were satisfied or very satisfied with the organization at baseline were less likely to reduce their FTE over the following months after controlling for sex, age, duration of employment, job category, site, and baseline FTE (OR, 0.73; 95% CI, 0.65-0.83; *P* < .001; Table 3). A dose response association was also found, with each 1-point increase in satisfaction with the organization at baseline associated with lower odds of an HCW reducing their work effort over the ensuing 24 months (OR, 0.83; 95% CI, 0.79-0.88; *P* < .001) (eTable 3 in the Supplement).

**Table 3.** Multivariable Analysis Exploring Association of Health Care Workers’ Baseline Satisfaction With the Organization and Reduction in Work Effort

Variable	Odds ratio (95% CI)	*P* value	Overall *P* value
Satisfaction^a^	0.73 (0.65-0.83)	NA	<.001
Men^b^	0.45 (0.39-0.53)	NA	<.001
Age, y			
<35	1 [Reference]	NA	
35-44	0.57 (0.49-0.65)	<.001	<.001
45-54	0.46 (0.39-0.53)	<.001	
55-64	0.63 (0.54-0.74)	<.001	
≥65	1.40 (0.94-2.06)	.09	
Duration of employment, y			
≤5	1 [Reference]	NA	
6-10	0.90 (0.79-1.02)	.10	.002
11-15	0.78 (0.66-0.91)	.002	
>15	0.77 (0.66-0.89)	<.001	
Job category			
Nurse	1 [Reference]	NA	
Administrative office support	0.17 (0.14-0.21)	<.001	<.001
Business professional	0.09 (0.07-0.12)	<.001	
Clinical office support	0.24 (0.20-0.30)	<.001	
Health care professional	0.39 (0.33-0.47)	<.001	
Service and support personnel	0.55 (0.47-0.63)	<.001	
Technician or technologist	0.34 (0.29-0.40)	<.001	
Baseline FTE, per 0.10-point increase^c^	1.18 (1.14-1.22)	NA	<.001

Abbreviations: FTE, full-time equivalent; NA, not applicable.

^a^ Compared with not satisfied at baseline.

^b^ Compared with women.

^c^ Work effort was measured in full-time equivalent units recorded in payroll records.

## Discussion

In this large prospective cohort study of HCWs, burnout and professional satisfaction were associated with changes in work effort, as measured by FTE from payroll records, over the ensuing 24 months. HCWs with burnout at baseline had higher odds of reducing their work effort over the ensuing 24 months. Individuals who were satisfied with the organization at baseline had lower odds of reducing their work effort over the ensuing 24 months. Furthermore, a dose-response association of reduction in work effort was found for burnout and satisfaction. Each 1 point higher score in emotional exhaustion or depersonalization was associated with higher odds of reducing FTE over 24 months, and a 1-point increase in satisfaction score was associated with lower odds of reducing FTE. These findings persisted after controlling for sex, age, length of employment, job category, and baseline FTE.

Approximately 1 in 12 HCWs reduced their work effort over 24 months. Within this cohort, burnout was associated with HCWs reducing their work effort. A similar association between burnout and reduction in clinical work hours has been reported in a study of physicians by Shanafelt et al.^14^ The cost is likely to be substantial for employers, since benefits and other costs (eg, space, equipment) stay the same or increase (eg, recruiting expenses) while revenue generated by the employee decreases. For example, the cost of reduction in work effort, as well as turnover, attributable to burnout has been estimated at more than $7600 per employed physician annually.^32^

More than 1000 nurses, representing 15% of the nurses in this cohort, reduced their work effort and did so by an mean of nearly 20%. In addition to burnout, a variety of other factors, including nurse shift work, salary levels, and workplace flexibility, may also contribute to nurses reducing their work effort. Given the critical role nurses have in direct patient care, the shortage of nurses,^33,34^ and the costs associated with training, recruitment, and retention of nurses,^35,36^ a deeper understanding of the factors contributing to nurses reducing their work effort and whether this varies by type of nurse (eg, registered nurse, licensed practical nurse), work location (eg, inpatient, outpatient), or other factors is needed to inform interventions.

Given the association between work hours and burnout,^2,3^ reducing work effort may be a useful strategy for individuals seeking relief from burnout symptoms. A 2016 study of physicians^15^ reported that among those who reduced their work effort, 50% experienced a decrease in their emotional exhaustion symptoms and 37% experienced a decrease in their depersonalization symptoms over the ensuing 2 years. However, this strategy was not useful for all individuals, since some physicians had no change in their burnout symptoms or, despite reducing their work hours, experienced worsened burnout symptoms, highlighting the urgent need to address the underlying factors contributing to unrelenting workplace stress.

HCWs, employers (eg, health care organizations), and others (eg, government regulators, health information technology companies, accreditors) have a shared responsibility to address system-level factors that may contribute to chronic workplace stress, lead to burnout, and hinder recovery. The 2019 consensus study report from the National Academies of Sciences, Engineering, and Medicine^2^ provides a road map for strategies to create positive work environments, reduce administrative burden, enable technology solutions, and provide support to HCWs. Evidence-based approaches are available to guide efforts.^37,38,39^

Strengths of this study include being prospective and including a large sample of HCWs working in diverse positions and practice settings. Additionally, we leveraged administrative and payroll data rather than relying on self-reported reduction in work effort. The survey response rate was high, and a validated metric was used to measure burnout.

### Limitations

This study has some limitations, including that all HCWs worked for the same organization. Although this may limit the generalizability of our findings, participants worked across geographic locations and within academic as well as community-based health care settings. In addition, despite being a longitudinal study, we are unable to determine causation. Furthermore, we measured a limited number of factors that were potentially associated with the decision to reduce work effort and additional factors were not included in the available data (eg, family obligations, socioeconomic circumstances, salary, local employment opportunities, changes in work schedule). Many of these variables are not routinely included in employer records, and prospective studies will be necessary to account for these variables in the future. Our sample includes HCWs in diverse practice settings (Olmsted County, Rochester, Minnesota; Duval County, Jacksonville, Florida; and, Maricopa County, Scottsdale and Phoenix, Arizona) with unemployment rates ranging from 2.7% to 4.3% and size of the workforce ranging from 85 538 to 2 146 260.^40^

## Conclusions

This cohort study found that among HCWs at a large, geographically distributed organization, burnout and professional satisfaction were associated with changes in work effort as measured by FTE levels from administrative and payroll records over the following 24 months. Additional research is needed to determine causal relationships among these factors and how best to improve the work environment to mitigate burnout and declines in professional satisfaction to lessen the risk of HCWs reducing their work effort.

## Notes

Supplement. **eTable 1.** Comparison of Participating and Nonparticipating Health Care Workers **eTable 2.** Emotional Exhaustion or Depersonalization at Baseline and Subsequent Reduction in Work Effort **eTable 3.** Satisfaction at Baseline and Subsequent Reduction in Work Effort
