# Supplementary material for: Characterization of Nonphysician Health Care Workers’ Burnout and Subsequent Changes in Work Effort
Source: JAMA Netw Open. 2021 Aug 20;4(8):e2121435. doi: 10.1001/jamanetworkopen.2021.21435 (PMC8379653; doi:10.1001/jamanetworkopen.2021.21435)
Supplement: Supplement. — eTable 1. Comparison of Participating and Nonparticipating Health Care Workers eTable 2. Emotional Exhaustion or Depersonalization at Baseline and Subsequent Reduction in Work Effort eTable 3. Satisfaction at Baseline and Subsequent Reduction in Work Effort [file jamanetwopen-e2121435-s001.pdf]

Supplemental Online Content

Dyrbye LN, Major-Elechi B, Thapa P, et al. Characterization of nonphysician health care workers' burnout and subsequent changes in work effort. *JAMA Netw Open*. 2021;4(8):e2121435. doi:10.1001/jamanetworkopen.2021.21435

eTable 1. Comparison of Participating and Nonparticipating Health Care Workers

eTable 2. Emotional Exhaustion or Depersonalization at Baseline and Subsequent Reduction in Work Effort

eTable 3. Satisfaction at Baseline and Subsequent Reduction in Work Effort

This supplemental material has been provided by the authors to give readers additional information about their work.

© 2021 Dyrbye LN et al. *JAMA Network Open.*

eTable 1. Comparison of Participating and Nonparticipating Health Care Workers

**2015 Survey**	Non-responder (N=4963)	Responder (N=26292)	p value
**Age**			<0.001
<35	1652 (33.3%)	6678 (25.4%)	
35-44	1262 (25.4%)	6541 (24.9%)	
45-54	1150 (23.2%)	7298 (27.8%)	
55-64	813 (16.4%)	5451 (20.7%)	
≥65	86 (1.7%)	323 (1.2%)	
Missing	0	1	
**Length of Service**			<0.001
≤5 years	1718 (34.6%)	8574 (32.6%)	
6-10 years	1052 (21.2%)	5327 (20.3%)	
11-15 years	856 (17.2%)	4273 (16.3%)	
>15 years	1337 (26.9%)	8117 (30.9%)	
Missing	0	1	
**Gender**			0.635
Female	3810 (76.8%)	20265 (77.1%)	
Male	1153 (23.2%)	6027 (22.9%)	
**Job Category**			<0.001
Nurses	1833 (36.9%)	6595 (25.1%)	
Admin Office Support	315 (6.3%)	2971 (11.3%)	
Business Professionals	268 (5.4%)	4953 (18.8%)	
Clinical Office Support	463 (9.3%)	2884 (11.0%)	
Healthcare Professional	351 (7.1%)	2423 (9.2%)	
Service/Support Personnel	1025 (20.7%)	2968 (11.3%)	
Technician/Technologist	708 (14.3%)	3498 (13.3%)	
**Assigned FTE**[1]			<0.001
< .01	299 (6.0%)	364 (1.4%)	
.01 - .49	52 (1.0%)	89 (0.3%)	
.50 - .74	707 (14.2%)	1717 (6.5%)	

© 2021 Dyrbye LN et al. *JAMA Network Open.*

.75 - .99	1865 (37.6%)	6484 (24.7%)	
1	2040 (41.1%)	17638 (67.1%)	
**2017 Survey**	**Non-responder (N=4855)**	**Responder (N=26303)**	**p value**
**Age**			<0.001
<35	1324 (27.3%)	5201 (19.8%)	
35-44	1314 (27.1%)	6661 (25.3%)	
45-54	1132 (23.3%)	6926 (26.3%)	
55-64	946 (19.5%)	6796 (25.8%)	
>=65	139 (2.9%)	719 (2.7%)	
**Length of Service**			<0.001
<=5 years	1290 (26.6%)	6280 (23.9%)	
6-10 years	1019 (21.0%)	5391 (20.5%)	
11-15 years	887 (18.3%)	4622 (17.6%)	
>15 years	1659 (34.2%)	10010 (38.1%)	
**Gender**			0.259
Female	3705 (76.3%)	20268 (77.1%)	
Male	1150 (23.7%)	6035 (22.9%)	
**Job Category**			<0.001
Nurses	1591 (32.8%)	5884 (22.4%)	
Admin Office Support	310 (6.4%)	2889 (11.0%)	
Business Professionals	288 (5.9%)	5309 (20.2%)	
Clinical Office Support	454 (9.4%)	2826 (10.7%)	
Healthcare Professional	631 (13.0%)	3229 (12.3%)	
Service/Support Personnel	892 (18.4%)	2656 (10.1%)	
Technician/Technologist	689 (14.2%)	3510 (13.3%)	
**Assigned FTE**[1]			<0.001
< .01	328 (6.8%)	302 (1.1%)	
.01 - .49	48 (1.0%)	59 (0.2%)	
.50 - .74	653 (13.5%)	1729 (6.6%)	

© 2021 Dyrbye LN et al. *JAMA Network Open.*

.75 - .99	1801 (37.1%)	6595 (25.1%)	
1	2025 (41.7%)	17618 (67.0%)	

[1] Employment status policy defines those as authorized to work 40 hours per week to be full-time (1 FTE). Those authorized to work 20-39 hours per week are classified as 0.75 to 0.99 FTE. Individuals authorized to work a regular schedule but less than 20 hours per week or 40 hours per pay period are classified as .01-.49 FTE and are part-time. Supplemental staff work a minimum of 96 hours per payroll year based on staffing needs.

© 2021 Dyrbye LN et al. *JAMA Network Open.*

eTable 2. Emotional Exhaustion or Depersonalization at Baseline and Subsequent Reduction in Work Effort

**Variable**	**Level**	**Odds Ratio (95% CI)**	**p-value**	**Overall p-value**
Emotional Exhaustion				
Emotional Exhaustion at baseline	For every one point increase in score	1.12 (1.10-1.16)		<.001
Gender	Male vs. Female	0.47 (0.40-0.54)		<.001
Age	<35	Reference		<.001
	35-44	0.59 (0.51-0.67)	<.001	
	45-54	0.50 (0.42-0.58)	<.001	
	55-64	0.69 (0.59-0.82)	<.001	
	>=65	1.54 (1.03-2.29)	.03	
Duration of employment	≤5 years	Reference		<.001
	6-10 years	0.87 (0.77-0.99)	.04	
	11-15 years	0.76 (0.65-0.90)	.001	
	>15 years	0.74 (0.64-0.86)	<.001	
Position	Nurses	Reference		<.001
	Admin Office Support	0.17 (0.14-0.22)	<.001	
	Business Professionals	0.09 (0.07-0.12)	<.001	
	Clinical Office Support	0.25 (0.21-0.30)	<.001	
	Healthcare Professional	0.39 (0.33-0.47)	<.001	
	Service/Support Personnel	0.55 (0.48-0.64)	<.001	
	Technician/Technologist	0.35 (0.30-0.41)	<.001	
Baseline FTE	For every .10 increase	1.17 (1.13-1.21)		<.001
**Depersonalization**				

© 2021 Dyrbye LN et al. *JAMA Network Open.*

Variable	Level	Odds Ratio (95% CI)	p-value	Overall p-value
Depersonalization at baseline	For every one point increase in score	1.10 (1.06-1.14)		<.001
Gender	Male vs. Female	0.46 (0.39-0.53)		<.001
Age	<35	Reference		<.001
	35-44	0.58 (0.50-0.66)	<.001	
	45-54	0.48 (0.41-0.56)	<.001	
	55-64	0.68 (0.58-0.80)	<.001	
	>=65	1.54 (1.04-2.29)	.03	
Duration of Employment	≤ 5 years	Reference		<.001
	6-10 years	0.88 (0.77-1.00)	.05	
	11-15 years	0.77 (0.65-0.90)	.001	
	>15 years	0.75 (0.65-0.88)	<.001	
Position	Nurses	Reference		<.001
	Admin Office Support	0.18 (0.14-0.22)	<.001	
	Business Professionals	0.09 (0.07-0.12)	<.001	
	Clinical Office Support	0.25 (0.21-0.30)	<.001	
	Healthcare Professional	0.40 (0.33-0.47)	<.001	
	Service/Support Personnel	0.55 (0.48-0.64)	<.001	
	Technician/Technologist	0.35 (0.30-0.41)	<.001	
Baseline FTE	For every .10 increase	1.18 (1.13-1.22)		<.001

© 2021 Dyrbye LN et al. *JAMA Network Open.*

eTable 3. Satisfaction at Baseline and Subsequent Reduction in Work Effort

Variable	Level	Odds Ratio (95% CI)	p-value	Overall p-value
Satisfaction at baseline	For every one point increase in score	0.83 (0.79-0.88)		<.001
Gender	Male vs. Female	0.46 (0.39-0.53)		<.001
Age	<35	Reference		<.001
	35-44	0.57 (0.49-0.65)	<.001	
	45-54	0.46 (0.40-0.54)	<.001	
	55-64	0.64 (0.54-0.74)	<.001	
	>=65	1.44 (0.97-2.12)	.07	
Duration of Employment	≤ 5 years	Reference		.003
	6-10 years	0.88 (0.78-1.01)	.06	
	11-15 years	0.76 (0.65-0.90)	<.001	
	>15 years	0.75 (0.65-0.88)	<.001	
Position	Nurses	Reference		<.001
	Admin Office Support	0.17 (0.14-0.21)	<.001	
	Business Professionals	0.09 (0.07-0.12)	<.001	
	Clinical Office Support	0.25 (0.20-0.3)	<.001	
	Healthcare Professional	0.39 (0.33-0.47)	<.001	
	Service/Support Personnel	0.54 (0.47-0.63)	<.001	
	Technician/Technologist	0.34 (0.29-0.40)	<.001	
Baseline FTE	For every .10 increase	1.18 (1.14-1.22)		<.001

© 2021 Dyrbye LN et al. *JAMA Network Open.*
